# Electrochemical Behavior of Clay-Based Nanocomposites in an Ion-Exchange Gel Membrane for Supercapacitor Applications

**DOI:** 10.3390/gels12070576

**Published:** 2026-06-29

**Authors:** Borislava Mladenova, Gergana Ivanova, Antonia Bakalova, Elefteria Lefterova, Antonia Stoyanova

**Affiliations:** Institute of Electrochemistry and Energy Systems “Acad. Evgeni Budevski”, Bulgarian Academy of Sciences, Acad. Georgi Bonchev Str., Block 10, 1113 Sofia, Bulgaria; borislava.mladenova@iees.bas.bg (B.M.); gergana.ivanova@iees.bas.bg (G.I.); antonia.bakalova@iees.bas.bg (A.B.); edl@iees.bas.bg (E.L.)

**Keywords:** K10 clay, supercapacitors, gel membrane, ion-exchange hydrogel, manganese oxides, silver nanoparticles

## Abstract

The development of low-cost, environmentally friendly, and electrochemically stable electrode materials remains a significant challenge for supercapacitors. In the present study, composite materials based on a montmorillonite K10 clay support were synthesized and characterized. Coconut shell-derived activated carbon, manganese dioxide (MnO_2_), and/or activated carbon (YP-80F) modified with silver nanoparticles were utilized as functional additives to the clay matrix. The aim of this work is to enhance the specific capacitance and electrochemical stability of the materials through a synergistic effect between these individual components. The novelty of this study lies in the integration of montmorillonite K10-based nanocomposites with an ion-exchange hydrogel membrane and in the investigation of the synergistic effects of different functional additives on the electrochemical performance of supercapacitors. The electrodes were fabricated using a casting method, while a commercial membrane, pre-soaked in a sodium sulfate solution, was employed as both separator and electrolyte. The membrane functions as an ion-exchange hydrogel, contributing to high ionic conductivity and reduced interfacial resistance. The electrochemical results indicate that the presence of additives significantly improves electron transport within the system, while the K10 clay support acts as a stable structural framework. The obtained results demonstrate the potential of clay-based nanocomposites integrated into gel-polymer systems for the development of efficient, low-cost, and environmentally friendly next-generation supercapacitors.

## 1. Introduction

The efforts to increase energy security and reduce pollution necessitate the urgent development of new, environmentally friendly, and cost-effective materials for application in electrochemical energy sources. In this regard, supercapacitors attract significant interest due to their ability to combine high power, fast charge-discharge cycles, and long operational lifetime [1,2,3]. In these systems, both the electrode materials and the membranes play an important role. For a material to be suitable, it must possess a number of properties, such as high electrical conductivity and an electrochemically active surface, stability under electrochemical and thermal loads, as well as good wettability [4].

Among the investigated materials, clay minerals, particularly montmorillonite, are attracting increasing interest due to their low cost, natural abundance in the environment, and compatibility with environmental requirements [5,6]. These materials are widely used in electrochemical systems, primarily as electrode substrates, as well as components of membranes or additives to electrolytes [7,8,9,10,11]. In the field of electrochemical systems, particularly in the context of supercapacitors, the incorporation of clay minerals into composite architectures has been the subject of extensive research, as it contributes to increased structural stability and improved ion transport [12]. The layered structure of these materials allows for the intercalation of various functional species, including conductive phases and metal nanoparticles. This makes them promising candidates for the design of composite electrodes. Furthermore, clay minerals provide a large specific surface area, mechanical stability, and can facilitate ion transport in composite architectures [5,13,14,15,16].

The growing interest in montmorillonite in contemporary research is associated both with its natural properties and the possibility of structural modification by obtaining nanoclays and organophilic derivatives. These approaches allow effective control over its structure and lead to the creation of modified clay composites with improved physicochemical properties, making them promising materials for various energy applications [17,18]. An example of clay modification is the work of Xu-Feng Luo et al., where montmorillonite was modified with Fe-intercalated structures for supercapacitors. The resulting material possessed excellent redox properties and high mechanical strength, leading to a significant improvement in the electrochemical performance of supercapacitors [19]. Another interesting study is that of Shanshan Zhang et al., in which montmorillonite was used as a reagent in the synthesis of rGO. The authors found that by adding minerals, the agglomeration of nanoparticles was controlled, the chemical composition of the surface was regulated, and the material was protected from structural degradation. In this way, the electrochemical performance of the capacitor was improved, a specific capacitance of 315 F/g was achieved, as well as a long cycle life of 10,000 cycles [20]. In a study by Nay Yee Win Zaw et al., montmorillonite was used as a micro-template in the synthesis of NiTe-containing nanocomposites for supercapacitors. The authors found that the resulting material exhibited excellent energy and mechanical properties due to its hierarchically porous structure that facilitates ionic transport [21]. In addition to montmorillonite, its modified forms, such as K10, are also of interest, which also find application in composite materials. One such example is the work of Sandipan Maiti et al., who demonstrated that the inclusion of this type of clay in a composite improves ionic transport, which is associated with the presence of surface functional groups and the developed porous structure [22]. In modern literature studies, there is a prevailing opinion that clays occupy an important place as matrices in the synthesis of electrode materials. Their layered structure limits the agglomeration of the active components and ensures their uniform distribution, while maintaining access to the porous structure during prolonged operation of the electrodes [23].

Despite a growing number of studies conducted on clay-based composites, their integration with ion-exchange gel-polymer electrolytes remains understudied, with no unified design strategy in place. In particular, the interaction between layered clay matrices and advanced gel electrolytes and its impact on ion transport and interfacial phenomena in electrochemical systems has not been systematically investigated.

In this context, the present study introduces a novel approach using montmorillonite K10 as a functional structural matrix to develop clay nanocomposite electrodes for gel-electrolyte supercapacitor systems. In contrast to most clay-based supercapacitors that have been described to date, where the focus has primarily been on the composition of the electrodes, this study investigates the interaction between clay-matrix nanocomposite electrodes and an Aquivion^®^ ion-conductive gel membrane within the same electrochemical system. Unlike conventional approaches, this study focuses on the targeted incorporation of multiple functional additives into the clay structure to modulate its electrochemical activity, surface properties, and charge storage mechanisms.

This work is focused on the integration of nanocomposite electrodes with an Aquivion^®^ ion-exchange gel membrane, forming a hybrid clay system for electrochemical energy storage. This enables synergistic interaction between the electrode architecture and the electrolyte. By establishing correlations between composition, structure, and electrochemical characteristics, the study provides further insight into the design principles of hybrid clay-gel systems. The results provide useful insight into the design of clay-based hybrid materials for sustainable supercapacitor applications.

## 2. Results and Discussion

### 2.1. Physicochemical Characterizations

The phase composition and structure of pure montmorillonite K10 and its modified composites were investigated using X-ray diffraction (XRD), while the elemental composition of the active additives was analyzed via X-ray fluorescence (XRF) and energy-dispersive X-ray spectroscopy (EDX).

**Figure 1:**
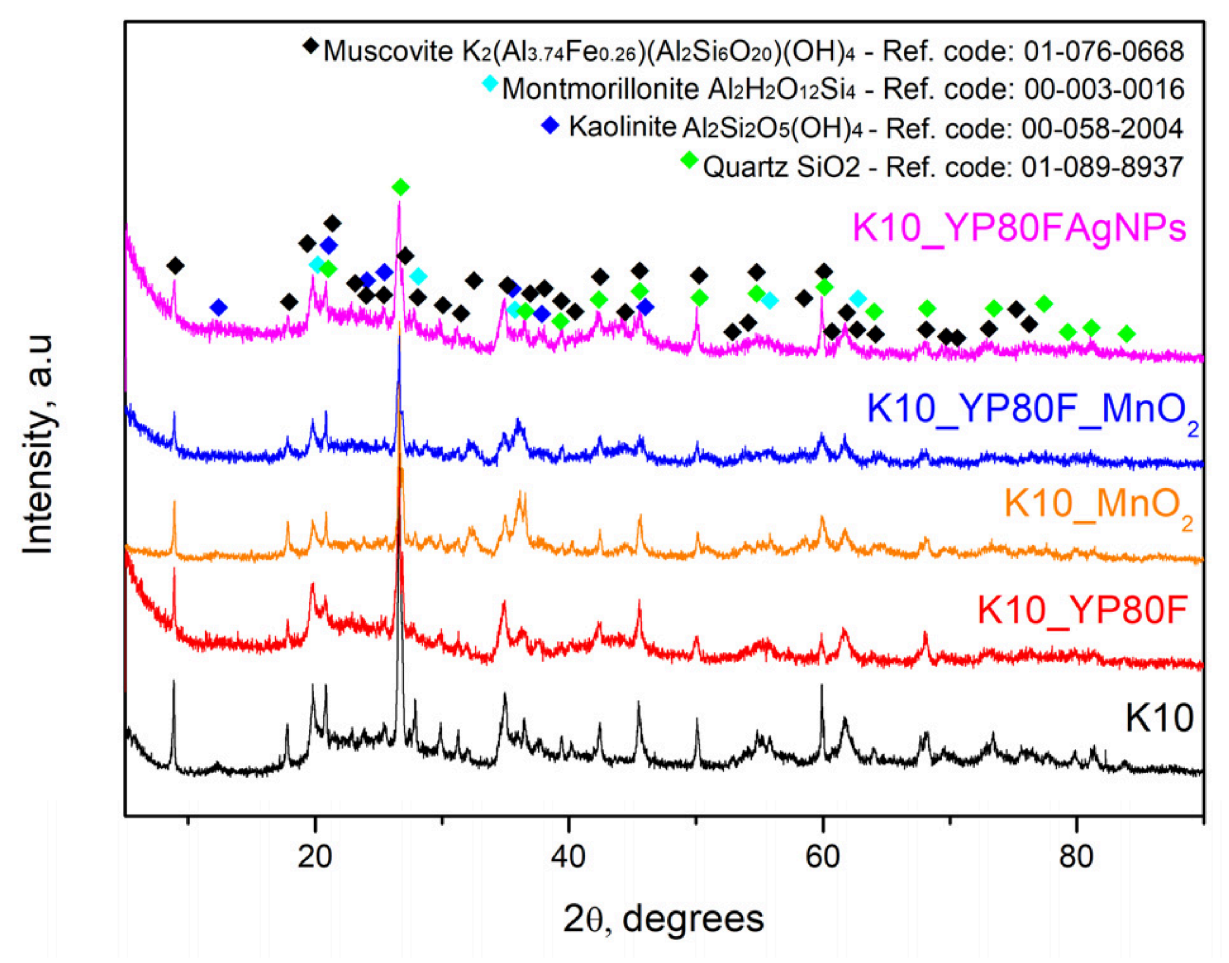
X-ray diffraction (XRD) patterns of montmorillonite (K10) and its modified counterparts, denoted as K10_YP80F, K10_MnO2, K10_YP80F_MnO_2_, and K10_YP80F_YP80FAgNPs.

Figure 1 shows the X-ray diffraction (XRD) patterns of pure K10 and its composites modified with activated carbon (YP-80F), as well as those of samples functionalized with MnO_2_ and doped with silver nanoparticles. The diffraction pattern of pure K10 is dominated by reflections originating from the acid-resistant mineral phases of quartz and muscovite, combined with an elevated background, suggesting the presence of both crystalline and partially amorphous regions. The most intense diffraction peak at 26.52° 2θ, corresponding to the (011) crystallographic plane of trigonal SiO_2_, indicates that quartz remains structurally unaffected by the aggressive acid activation treatment applied during the manufacture of commercial K10 montmorillonite. The other acid-resistant phase is muscovite, identified by its characteristic peaks at 35.03° 2θ, where the (131) and (−202) reflections overlap, and at 8.86° 2θ, corresponding to the (002) reflection with an interplanar spacing of d = 9.97 Å. The absence of the characteristic basal reflection of montmorillonite at 6.31° 2θ (d_(001)_ = 14 Å) indicates that a substantial loss of long-range structural order has been achieved. However, the weak reflection observed at 20.12° 2θ suggests that the phase has not become completely X-ray amorphous. Kaolinite has also undergone significant structural disordering, as evidenced by the weakly expressed (001) basal reflection at 12.33° 2θ, which indicates disordered stacking of its layers. The incorporation of functional additives into the clay matrix results in a slight shift in the muscovite (002) reflection toward higher diffraction angles (8.86° → 8.92° 2θ), corresponding to a decrease in the interlayer spacing from 9.97 Å to 9.91 Å. This shift is likely associated with structural rearrangement, partial dehydration, lattice strain, or interfacial interactions between muscovite and the deposited MnO_2_/Ag/carbon particles.

Following the incorporation of YP-80F carbon, a decrease in peak intensity is observed, along with the complete disappearance of some reflections, which may be attributed to additional amorphization of the K10-YP80F composite.

A comparison between the MnO_2_-containing composite materials (K10_MnO_2_ and K10_YP80F_MnO_2_) provides further insight into the structural characteristics of the manganese-modified composites. Although XRF analysis (Table 1) confirms a high manganese content of around 41–42% in both cases, the XRD patterns do not exhibit any well-defined reflections corresponding to MnO_2_ crystalline phases. Instead, only a broad, subtle hump is visible at the expected 37.52° 2θ positions for the main MnO_2_ reflections. Furthermore, it is observed that the K10_YP80F_MnO_2_ sample exhibits a higher background at low 2θ angles compared to K10_MnO_2_. This increase is likely attributed to the incorporation of YP-80F into the K10 matrix. This suggests that the manganese phase is likely present in a finely dispersed or amorphous state, consisting of crystallites too small to be phase-separated. Similarly, the X-ray diffraction pattern of the K10_YP80FAgNPs composite does not show distinct silver reflections, but X-ray fluorescence analysis confirms a silver content of 0.54 wt.%. The absence of detectable peaks is consistent with such a low concentration, which likely falls below the detection limit of the XRD technique.

The absence of well-defined peaks in the XRD patterns of the MnO_2_ and Ag-containing composites, despite their confirmation via XRF analysis, suggests a confining effect of the K10 clay matrix. This can be attributed to the nanosized MnO_2_ and Ag particles and their partial overlap with K10 diffraction peaks, as well as to the layered clay structure, which restricts cluster formation and promotes a high dispersion of the additives. Consequently, the structural synergy of this configuration is expected to facilitate rapid ionic transport and enhance pseudocapacitive behavior.

**Figure 2:**
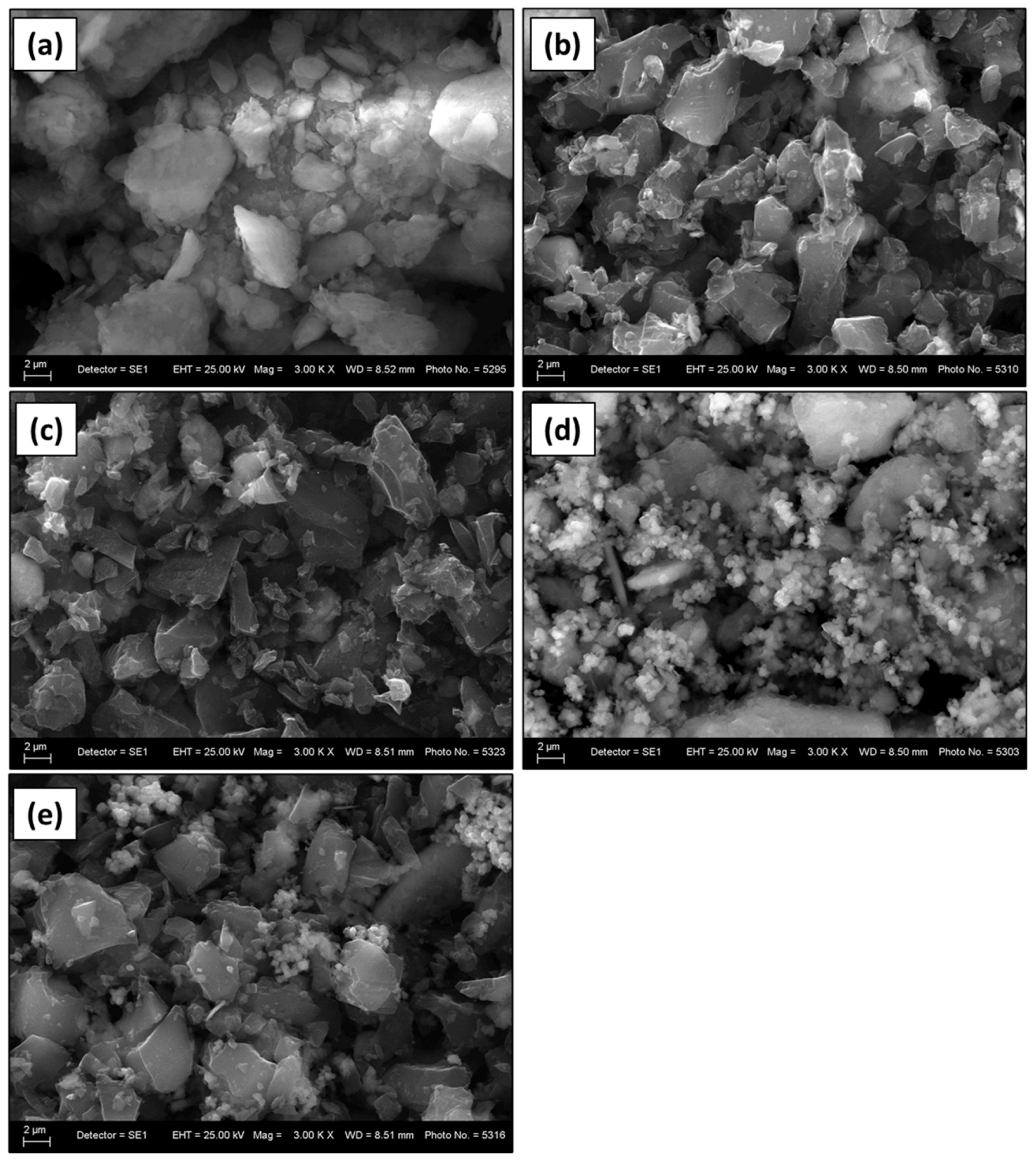
SEM micrographs of: (**a**) pure montmorillonite K10; (**b**) K10_YP80F; (**c**) K10_YP80FAgNPs; (**d**) K10_MnO_2_; and (**e**) K10_YP80F_MnO_2_.

**Figure 3:**
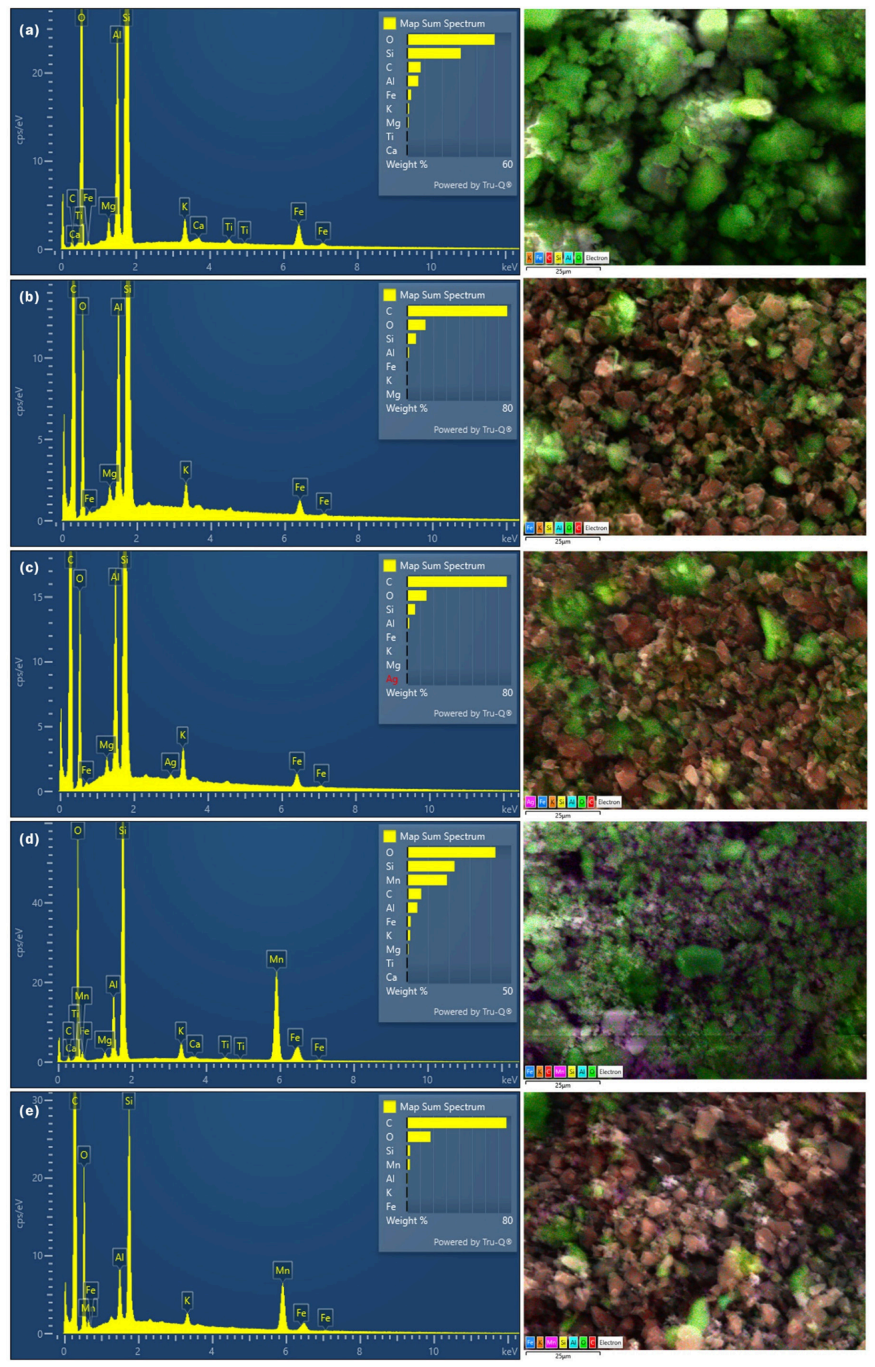
EDX mapping and corresponding quantitative elemental analysis (wt.%) of: (**a**) pristine montmorillonite K10; (**b**) K10_YP80F; (**c**) K10_YP80FAgNPs; (**d**) K10_MnO_2_; and (**e**) K10_YP80F_MnO_2_.

The SEM micrographs (Figure 2) illustrate the structural evolution of Montmorillonite K10 clay through various modification steps involving activated carbon (YP80F), silver nanoparticles (AgNPs), and manganese dioxide (MnO_2_). Untreated K10exhibits the typical lamellar structure of montmorillonite: stacked, platelet-like particles with irregular interparticle voids and sharp-edged aggregates. When YP-80F is added, the clay lamellae remain intact but become partially covered by an amorphous carbon phase. This phase smooths the particle edges and contributes to the formation of a more interconnected porous network. In systems containing MnO_2_, discrete oxide domains are distributed across the clay surface, with periodic local agglomeration of the metal-oxide phase. When YP-80F and MnO_2_ are introduced simultaneously, the carbon phase binds the clay lamellae together and partially covers the manganese-containing regions. This leads to a more continuous hybrid architecture. In the Ag-containing composite, the individual nanoparticles cannot be clearly distinguished by SEM due to their low concentration and fine dispersion in the matrix. However, their presence is confirmed by elemental mapping (Figure 3) and EDX analysis (Table 2). Overall, the observed morphology confirms that K10 montmorillonite acts as a stable structural matrix that limits the agglomeration of the active phases and facilitates their uniform distribution across the composite surface, which is in good agreement with the XRD analysis.

To further evaluate the textural properties of the synthesized composites, nitrogen adsorption-desorption measurements were performed, and the specific surface area was determined using the BET method. Measurements of nitrogen adsorption revealed significant differences in the specific surface area of the materials studied. Untreated K10 had a BET surface area of approximately 230 m^2^ g^−1^, while the addition of activated carbon increased the surface area to 1135 m^2^ g^−1^ for K10_YP80F. The K10_MnO_2_ sample exhibited a surface area of 62 m^2^ g^−1^, wheres the K10_YP80F_MnO_2_ composite retained a high surface area of 1009 m^2^ g^−1^). The K10_YP80FAgNPs exhibited the highest value (1206 m^2^ g^−1^). These results indicate that the addition of activated carbon and functional additives preserves a highly developed porous structure, which corresponds to the improved electrochemical properties observed in the multicomponent composites.

### 2.2. Electrochemical Results

Cyclic voltammetry (CV), galvanostatic charge-discharge (GCD), and electrochemical impedance spectroscopy (EIS) were used to evaluate the electrochemical behavior of the resulting clay-based hybrid electrodes, in order to clarify the influence of the individual functional additives on charge storage and transport processes.

**Figure 4:**
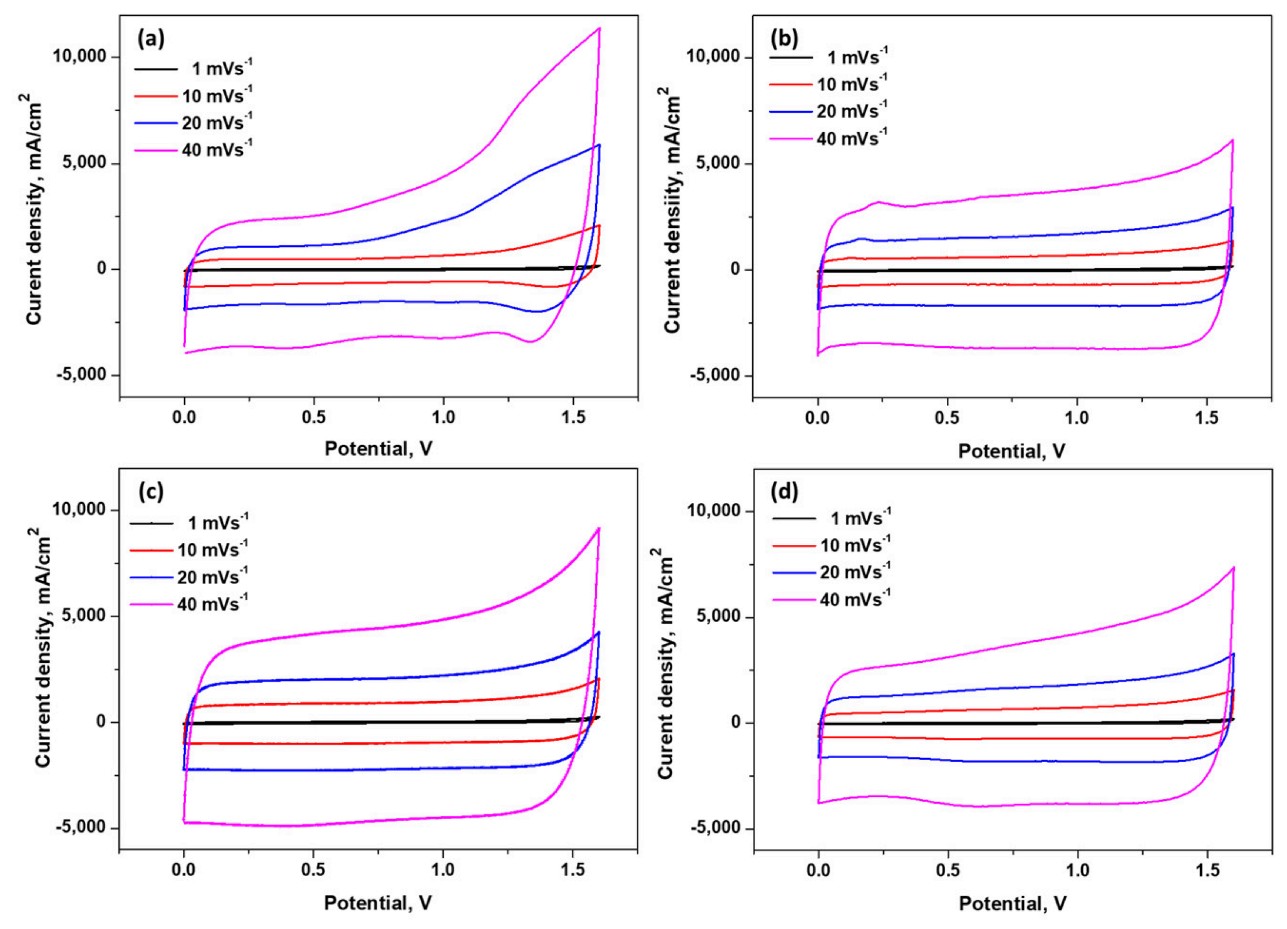
Cyclic voltammograms profiles of: K10_YP80F_MnO_2_ (**a**); K10_YP80FAgNPs (**b**); K10_YP80F (**c**); K10_MnO_2_ (**d**).

Figure 4 shows the cyclic voltammograms of the clay-based composite materials. All measurements were performed within a potential window from 0 to 1.6 V at scan rates ranging from 1 to 40 mV/s. For all samples, an increase in current density was observed with increasing scan rate. For the composite material containing silver nanoparticles (Figure 4b), as well as for the K10_YP80F sample containing only activated carbon (Figure 4c), the shape of the curves is close to rectangular, which is characteristic of capacitive behavior. In contrast, materials containing manganese dioxide (K10_YP80F_MnO_2_, see Figure 4a, and K10_MnO_2_, see Figure 4d) deviate noticeably from the ideal rectangular shape and exhibit more pronounced current peaks. These characteristics suggest the presence of additional Faradaic processes. More specifically, this behavior is attributed to pseudocapacitive charge storage resulting from the fast, reversible redox reactions of MnO_2_, which introduce a combination of capacitive and diffusion-controlled mechanisms.

**Figure 5:**
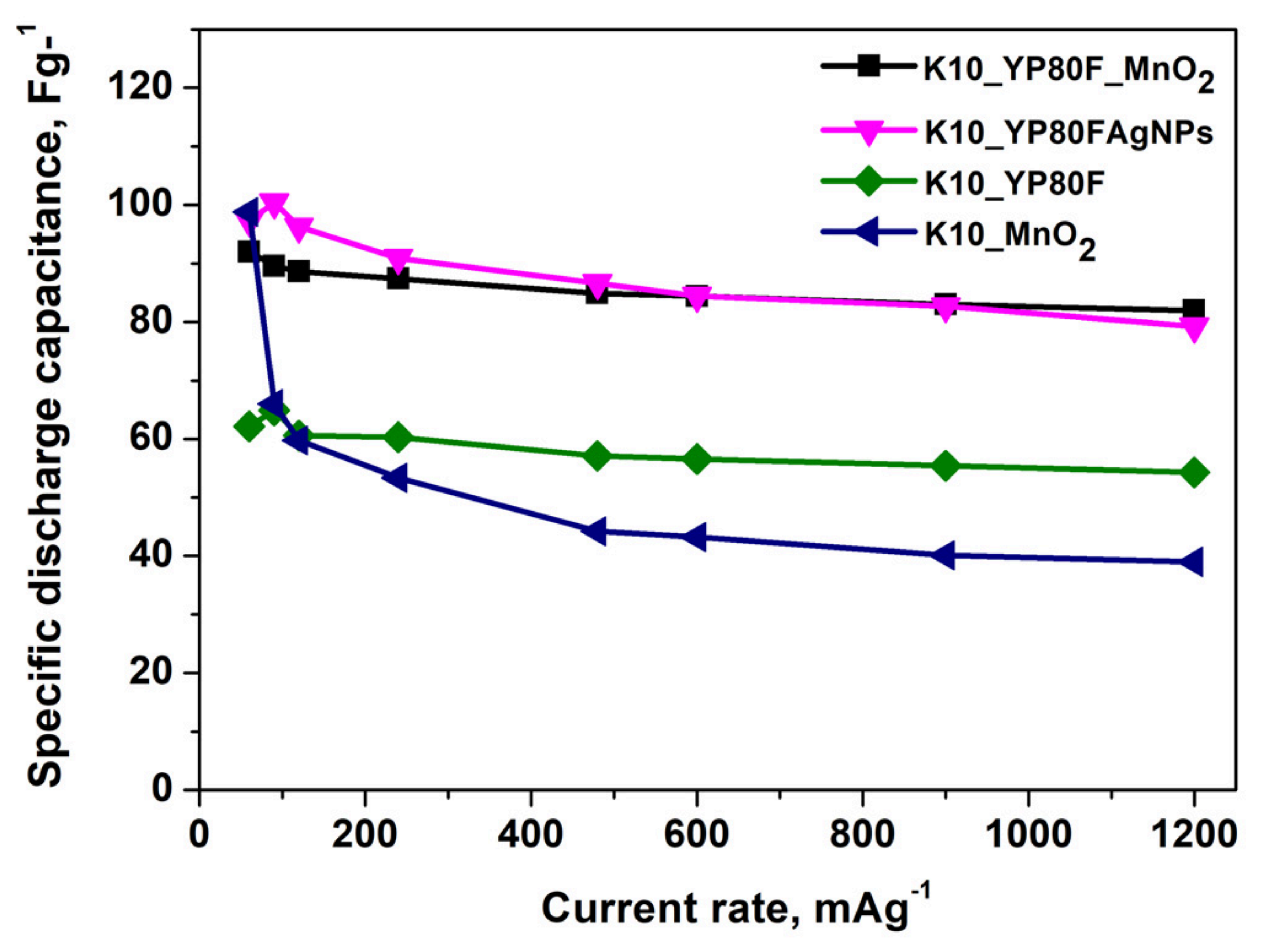
Specific discharge capacitance of the composite materials: K10_YP80F; K10_YP80FAgNPs; K10_MnO_2_; and K10_YP80F_MnO_2_.

The practical charge-storage capability of the composites is further reflected by the specific discharge capacitance values shown in Figure 5.

The results clearly show that systems consisting of a single MnO_2_ or activated carbon component exhibit relatively low capacitance values of ~60 F g^−1^, indicating limited charge storage efficiency when incorporated individually into the K10 matrix. In contrast, multicomponent composites demonstrate improved capacitance and stability at higher current densities. The K10_YP80F_MnO_2_ electrode maintains a capacitance of 92–82 F g^−1^, while the K10_YP80FAgNPs electrode retains a capacitance of 97–79 F g^−1^. These values demonstrate an enhanced rate capability compared to that of single-phase systems. This trend is consistent with the higher current response observed in the corresponding CV profiles (Figure 4a,b), which suggests improved electrochemical kinetics in the hybrid architectures. Such performance is associated with the combined contribution of the conductive (AgNPs), redox-active (MnO_2_), and carbon components, which support more efficient charge transport within the clay-based network. In contrast, the MnO_2_-only system exhibits reduced rate performance, demonstrating a significant capacitance decrease at 60 mA g^−1^ despite an initial value of ~100 F g^−1^. This may be due to the limited electronic conductivity of MnO_2_ and the intrinsic transport constraints of the K10 matrix, which restrict the utilization of active sites under higher current loads. Overall, the results suggest that charge storage performance benefits from the presence of multiple complementary components, whereas single-phase systems are more strongly limited by the properties of the clay matrix.

**Figure 6:**
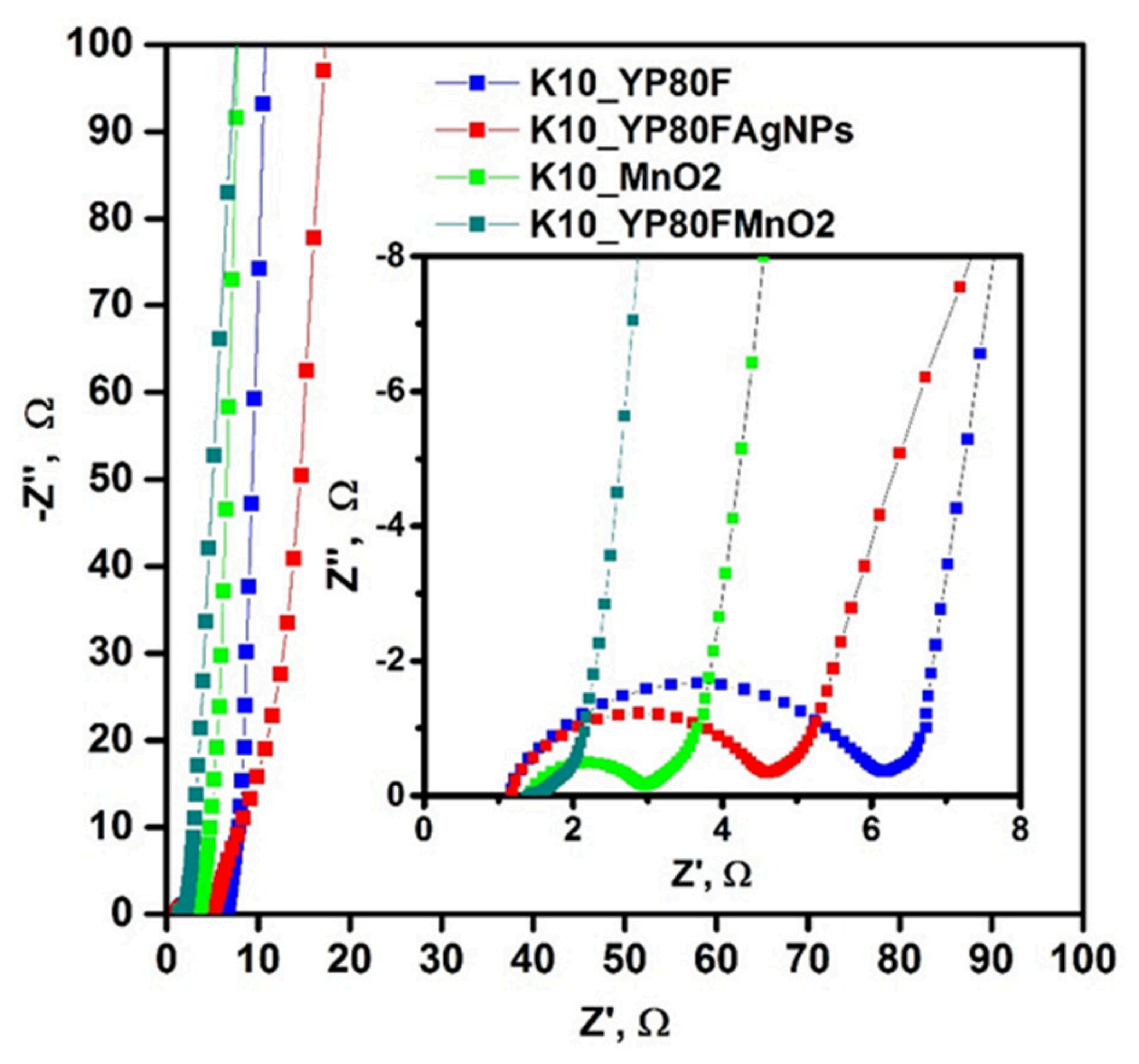
Nyquist plots of cells with K10_YP80F, K10_YP80FAgNPs, K10_YP80F_MnO_2_, and K10_MnO_2_ electrodes.

The incorporation of montmorillonite K10 has a dual effect on the electrochemical characteristics of the systems under study. On the one hand, its layered, porous structure promotes electrolyte absorption and facilitates ion incorporation into the membrane matrix. However, the clay phase’s inherent low electronic conductivity can lead to additional resistance, which impedes charge transfer processes to some extent. This trend is confirmed by the Nyquist plots obtained from electrochemical impedance spectroscopy (EIS) measurements of the assembled supercapacitors (see Figure 6).

**Scheme 1:**
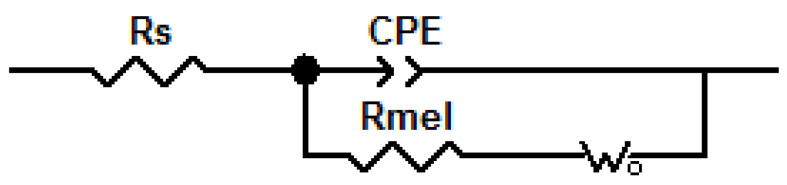
Modified Randles equivalent circuit.

The Nyquist plots of the investigated electrodes exhibit the characteristic behavior of porous materials. The modified Randles equivalent circuit (Scheme 1) is used for analyzing and fitting (program ZView 3.2b).

The obtained parameters are shown in Table 3. In this circuit, Rs includes the ohmic resistance of the electrolyte, electrical contacts, and current collectors. The Rmel-CPE element describes the semicircle observed in the high to medium-frequency region and reflects the coupled electronic and ionic transport through the porous electrode material. The Warburg Open (Wo) element, also known as the Generalized Finite Warburg (GFW) element, is characterized by the parameters Rd (diffusion resistance), WoT (time constant), and Wo-P (exponent ≤ 0.5). It is used to model finite-length ion diffusion within the porous electrode structure.

The fitted parameters reveal only minor differences in the serial resistance (Rs) for all investigated electrodes. The Rmel values suggest significant differences in the electronic and ionic transport properties of the electrode materials, with K10_YP80F_MnO_2_ exhibiting the lowest resistance. The Wo-P parameter remains close to 0.5 for all samples, confirming Warburg-type diffusion behavior. Differences in Rd and Wo-T indicate variations in ion transport kinetics within the porous structure. The lowest time constant and Rd obtained for K10_YP80FAgNPs (0.05 s), followed by K10_YP80F_MnO_2_ (0.16 s), suggesting faster ion transport and shorter diffusion paths in these materials. In contrast, K10_YP80F and K10_MnO_2_ exhibited higher Wo-T values (0.28 and 0.23 s, respectively), indicating slower diffusion processes within the porous network.

The results indicate that the inclusion of MnO_2_ effectively compensates for the resistive effect of the clay-containing matrix. Compared to YP80F-based systems previously studied without K10, the present composites demonstrate that introducing montmorillonite leads to a trade-off between improved ionic accessibility and reduced electron transport, as reflected in the measured capacitance values [24].

The better capacitance stability at increasing current rate is probably due to the ability of the K10 montmorillonite matrix to retain residual moisture and electrolyte in its porous structure, which ensures efficient ion transport in the gel-electrolyte system even under intensive operating conditions. Furthermore, the interaction between the K10 matrix and the Aquivion^®^ ion-exchange gel membrane enhances interphase ionic mobility even further. This reduces charge transfer resistance and facilitates more efficient ion migration across the electrode-electrolyte interface, improving the system’s overall ionic conductivity. The effects of interactions between clay and polymer electrolyte on ion transport and electrochemical characteristics have previously been reported in layered hybrid systems and ionogel-based supercapacitors [25].

**Figure 7:**
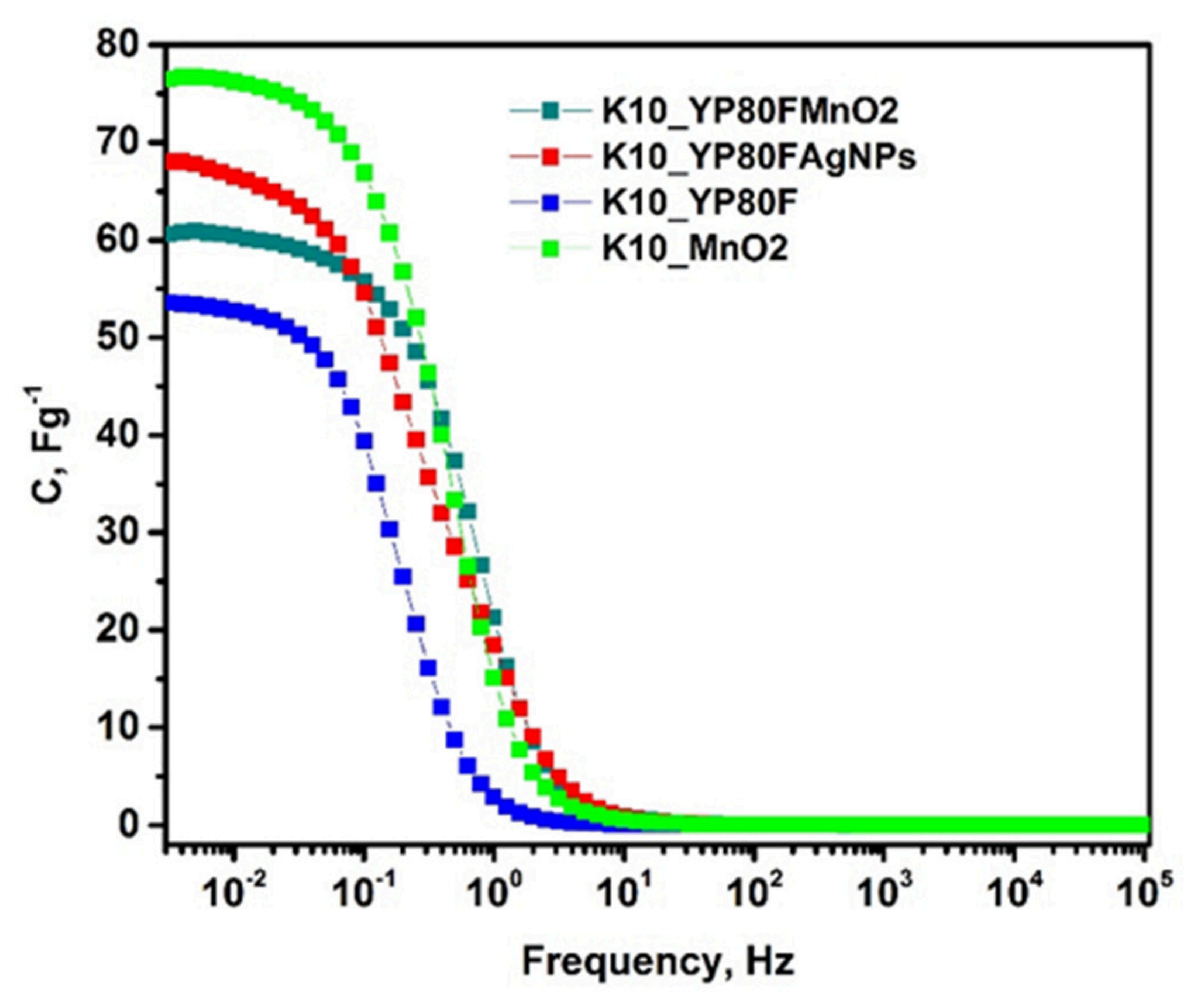
Specific capacitance vs. frequency of cells with K10_YP80F, K10_YP80FAgNPs, K10_YP80F_MnO_2_, and K10_MnO_2_ electrodes.

Figure 7 presents the specific capacitance versus frequency of cells with K10_YP80F, K10_YP80FAgNPs, K10_YP80F_MnO_2_, and K10_MnO_2_ electrodes, as derived from EIS measurements. Notably, despite the lower absolute values, the trend in specific capacitance is the same as that obtained at low current rates: the highest capacitance (73 F g^−1^) is exhibited by K10_MnO_2_, followed by K10_YP80FAgNPs, whereas the lowest value (51.5 F g^−1^) is observed for K10_YP80F.

**Figure 8:**
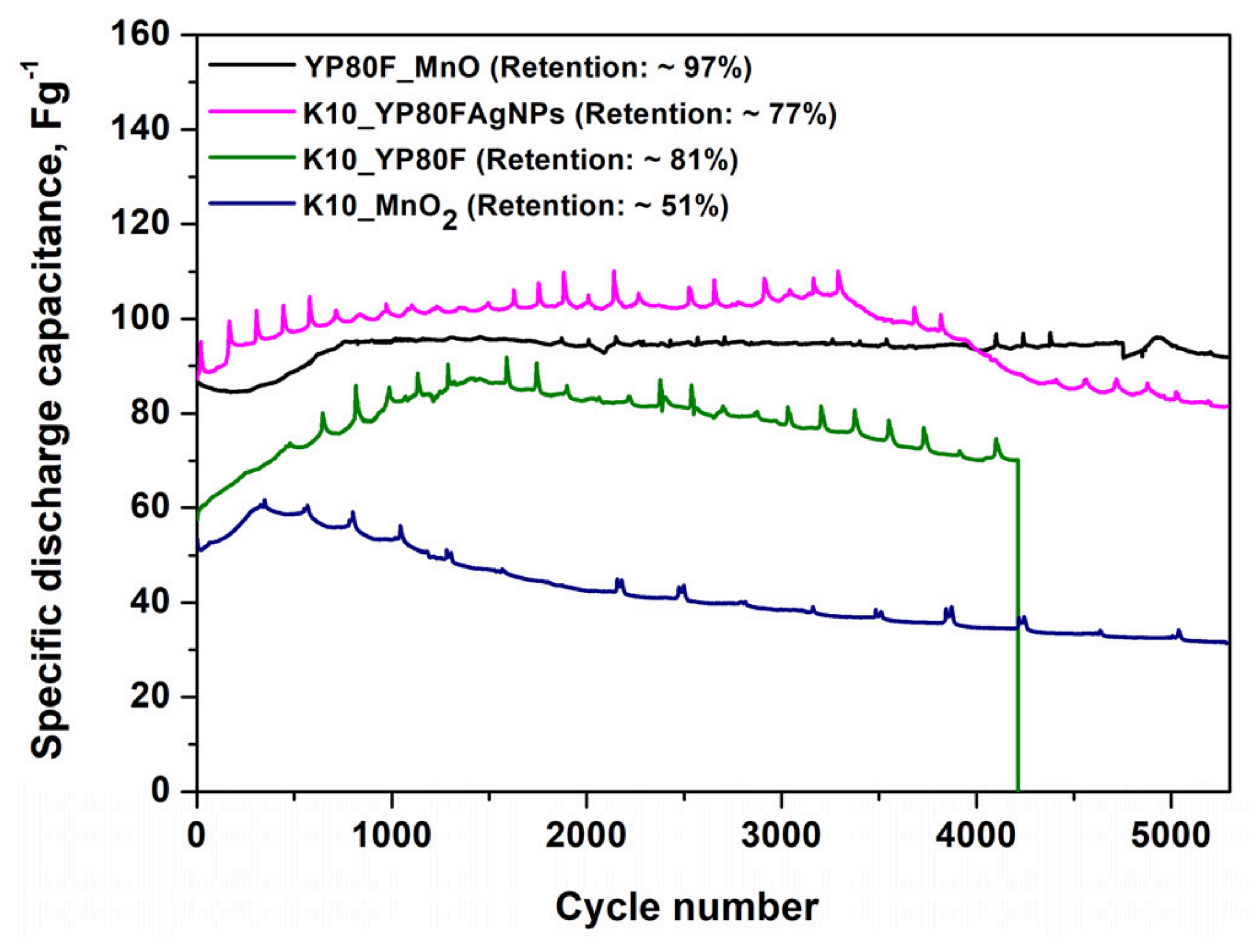
Long-term stability test of: K10_YP80F; K10_YP80FAgNPs; K10_MnO_2_ and K10_YP80F_MnO_2_.

The enhanced electrochemical stability is further supported by long-term cycling tests conducted at a current density of 240 mA g^−1^ (Figure 8), where all composites demonstrate sustained performance over extended operation, confirming their structural and electrochemical robustness. The ternary composite systems (K10_YP80F_AgNPs and K10_YP80F_MnO_2_), as well as the K10_MnO_2_ sample, maintained stable electrochemical performance over approximately 5000 charge–discharge cycles, whereas the K10_YP80F composite exhibited stable behavior over approximately 4000 cycles. This stabilizing effect is particularly evident in multicomponent systems, where pseudocapacitive and interfacial processes occur during repeated cycling without significant degradation in performance.

**Figure 9:**
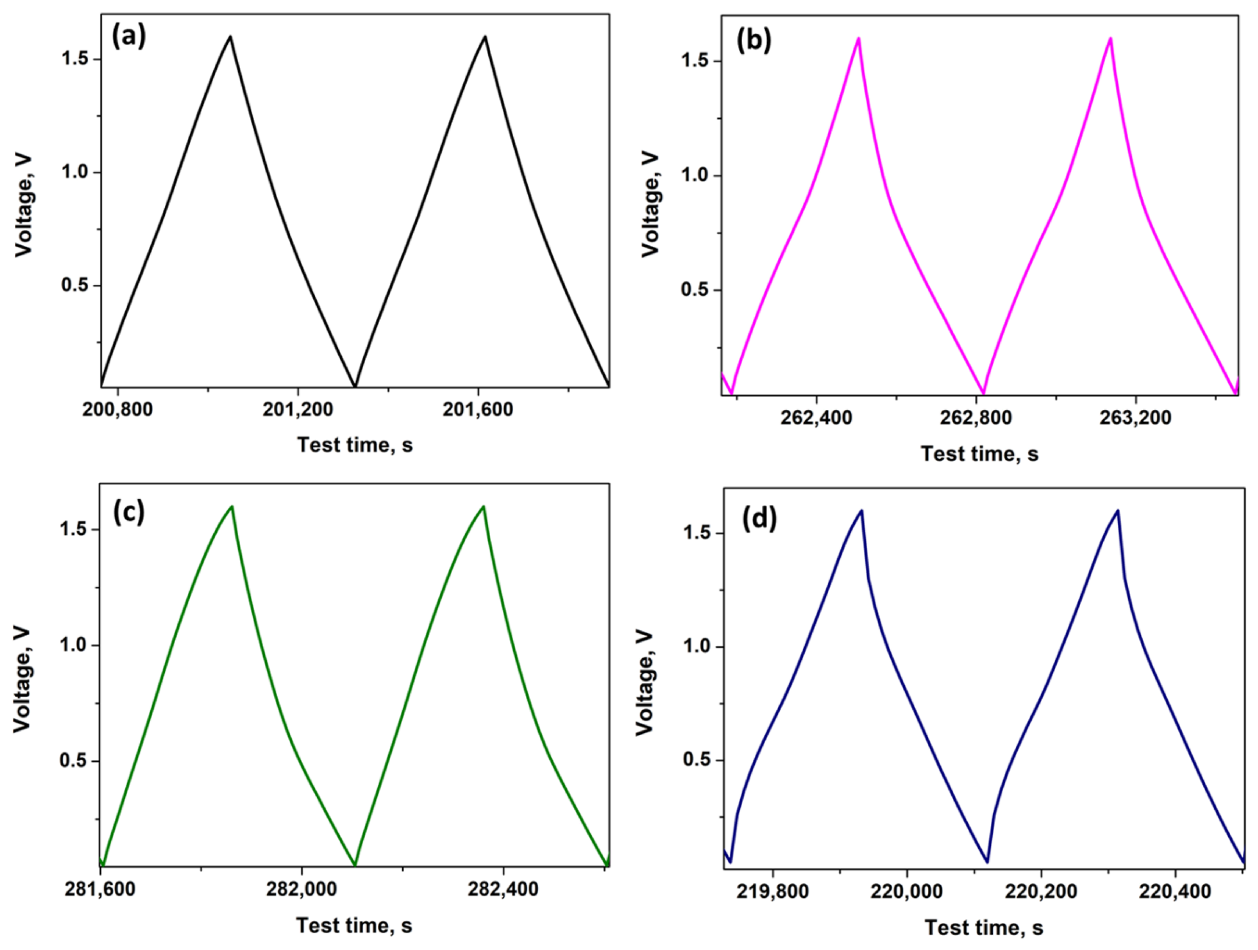
GCD profiles of: K10_YP80F_MnO_2_ (**a**), K10_YP80FAgNPs (**b**), K10_YP80F (**c**); K10_MnO_2_ (**d**).

The GCD profiles at 240 mA g^−1^ (Figure 9) are triangular and symmetrical, indicating capacitive behavior and good reversibility. The K10_YP80FAgNPs composite has the longest discharge time, reflecting its highest capacitance, due to improved conductivity from the AgNPs. The K10_YP80F_MnO_2_ sample also demonstrates improved characteristics, due to the pseudocapacitive contribution of MnO_2_, although these are limited by its lower conductivity. By contrast, the K10_YP80F and K10_MnO_2_ samples exhibit shorter discharge times, indicating reduced capacitance owing to limited electron transport. This behavior is consistent with the synergistic effects observed in the CV and capacitance results, confirming the role of additives in improving charge retention. Furthermore, montmorillonite K10, in combination with the ion-conductive Aquivion^®^ membrane, forms an ionogel-like system that provides efficient ion pathways and stable interfaces, thereby contributing to improved rate capability and electrochemical stability [25].

Overall, the electrochemical results suggest that montmorillonite K10 does not directly enhance capacity but rather acts as a structural ionic scaffold whose effectiveness strongly depends on the nature of the incorporated functional additives. Combining the clay matrix with only a single active component partially restricts charge transport due to its insulating nature. However, introducing conductive (AgNPs or YP80F) and pseudocapacitive (MnO_2_) domains in a complementary manner significantly improves the balance between ionic accessibility, electron penetration, and reversible charge storage.

**Figure 10:**
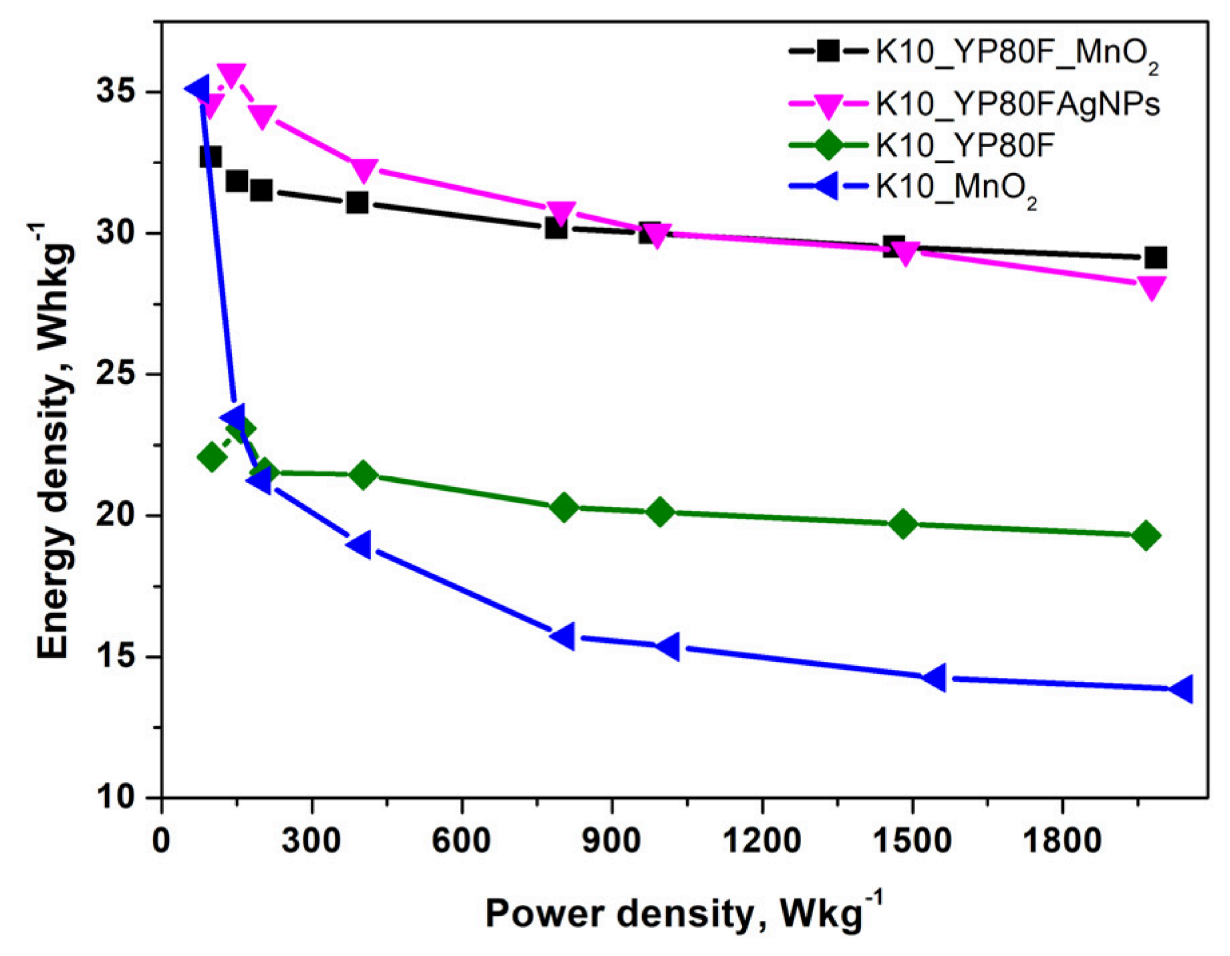
Ragone plots of: K10_YP80F_MnO_2_, K10_YP80FAgNPs, K10_YP80F; K10_MnO_2_.

Figure 10 presents the Ragone plots of the investigated composite electrodes, illustrating the relationship between energy density and power density for the different material systems. The obtained results provide additional insight into the electrochemical performance trends discussed above, confirming the superior behavior of the multi-component electrode architectures. In particular, the K10_YP80F_AgNPs and K10_YP80F_MnO_2_ composites exhibit the most favorable balance between energy and power characteristics. These materials maintain an energy density exceeding 29 Wh kg^−1^ at a power density of approximately 1500 W kg^−1^, highlighting their potential for high-performance energy storage applications. In contrast, the single-component electrodes demonstrate lower energy density values across the investigated power density range, indicating the beneficial effect of combining multiple active components within the electrode structure. Overall, the incorporation of multi-component electrode systems based on K10 clay in combination with the Aquivion^®^ membrane contributes to improved electrochemical performance and a more stable energy–power relationship, which is highly relevant for the design of efficient and durable energy storage devices.

## 3. Conclusions

This study used K10 montmorillonite as a multifunctional structural matrix to develop electrode materials containing various functional additives in combination with an Aquivion^®^ gel membrane. The results demonstrate that the layered structure of the clay enables the functional phases to be distributed uniformly while maintaining a porous network structure that is accessible to electrolyte ions. This structural organization directly influences the electrochemical behavior of the system by ensuring efficient ion transport. Combining K10-based electrodes with the ion-conductive Aquivion^®^ membrane creates a hybrid clay-polymer architecture. This configuration provides a favorable environment for charge storage and transport processes during electrochemical cycles. The electrochemical characteristics are determined by the interaction between the interface (where the composite electrode meets the Aquivion^®^ gel membrane) and the rapid electron transport provided by the conductive additives. Notably, three-component materials exhibit significantly higher capacity and improved cycling stability compared to single-component systems. Specifically, single-component systems exhibit relatively low capacitance values of approximately 60 F g^−1^, whereas multicomponent composites demonstrate improved performance. For example, K10_YP80F_MnO_2_ maintains 92–82 F g^−1^ and K10_YP80FAgNPs retain 97–79 F g^−1^ at different current rates, together with excellent structural and electrochemical stability during long-term cycling at 240 mA g^−1^. Additionally, they exhibit energy densities exceeding 29 Wh kg^−1^ at a power density of 1500 W kg^−1^, highlighting their potential for use in high-performance energy storage applications.

In summary, the results confirm that montmorillonite K10 is an effective structural matrix for constructing hybrid electrode systems. It provides mechanical stability and high ionic accessibility while improving electrochemical performance. Furthermore, when integrated with the Aquivion^®^ gel membrane, it results in a well-defined hybrid electrode-membrane architecture that enables effective coupling of ionic and electronic transport.

## 4. Materials and Methods

### 4.1. Materials

Montmorillonite K10 (Sigma-Aldrich, St. Louis, MO, USA) was used as the clay carrier in this work. The following functional additives were incorporated into the clay matrix: pre-synthesized MnO_2_, obtained by chemical precipitation method, described in detail in our previous publication [9]; activated carbon modified with silver nanoparticles, synthesized by the method presented in our publication [26]; as well as commercial activated carbon from coconut shells (YP-80F Kuraray Co., Ltd. Tokyo, Japan). The incorporation of the additives into the clay matrix was carried out by a two-step method using ultrasound. K10 was dispersed in isopropyl alcohol, and the resulting suspension was treated in an ultrasonic bath for 10 min. Then, a predetermined amount of the respective additive was added to it, and the suspension was subjected to additional ultrasonic treatment for another 10 min. The aim was to achieve better homogenization between the clay and the additives, as well as deeper penetration and effective fixation of the active components in the structure of the clay carrier. The resulting suspensions were left to stand for 24 h at room temperature, after which the samples were dried in a air-drying oven at 40 °C for 6 h. The electrodes were prepared using the casting method. A homogeneous paste was prepared containing 75 wt.% nanocomposite material, 10 wt.% graphite (ABG 1005 EG1, Superior Graphite, Chicago, IL, USA), 5 wt.% carbon fibers, and 10 wt.% polyvinylidene difluoride (PVDF) as a binder, dissolved in N-methyl-2-pyrrolidone (NMP) [27]. The resulting paste was applied as a thin layer on a glass substrate. After drying at 70 °C, the elastic layer was separated from the substrate and subjected to additional heat treatment.

### 4.2. Physicochemical Characterization of Materials

X-ray diffraction (XRD) analysis was employed to determine the phase composition of the synthesized materials. Measurements were taken using a Philips PW 1030 diffractometer (Philips Analytical, Almelo, the Netherlands) with Cu Kα radiation (λ = 1.5406 Å) in θ–2θ Bragg–Brentano geometry. Diffraction patterns were recorded within the 2θ range of 5–90°, with an increment of 0.02° between steps and a counting time of 1 s per step. Phase identification was performed using the PDF-2 database (ICDD, 2022). The elemental composition of the samples was determined by energy-dispersive X-ray fluorescence (EDXRF) spectroscopy. The measurements were carried out using an Epsilon 1 spectrometer (Malvern Panalytical, Malvern, UK), equipped with a silver anode X-ray tube operating at up to 50 kV and a maximum power of 10 W. The instrument enables the detection of elements in the range from sodium (Na) to americium (Am). The quantitative elemental composition obtained from the analysis is presented in weight percent (wt.%). The surface morphology and elemental composition of the samples were investigated using a Zeiss Evo 10 scanning electron microscope (Carl Zeiss Microscopy, Oberkochen, Germany). This system was integrated with an Oxford Ultim Max 40 EDX detector (Oxford Instruments, Abingdon, UK). Data acquisition and analysis were performed via Smart SEM (V07.05) and AZtec (version 6.1 HF4) software, respectively. The textural properties of the synthesized composite materials were investigated by nitrogen adsorption–desorption measurements using a Quantachrome AutoSorb iQ analyzer (Quantachrome Instruments Boynton Beach, FL, USA). The specific surface area was determined according to the Brunauer–Emmett–Teller (BET) method.

### 4.3. Electrochemical Characterization

The electrochemical characteristics were studied in an asymmetric configuration, implemented in a Swagelok-type cell. As a separator between the two electrodes, an Aquivion^®^ E87–05S (Sigma-Aldrich, Milan, Italy) membrane soaked in a Na_2_SO_4_ solution, operating as an ion-exchange gel separator, was used. Cyclic voltammetry (CV) and electrochemical impedance spectroscopy (EIS) were performed with a Multi PalmSens 4 potentiostat. CV measurements were performed at scan rates from 1 to 40 mV s^−1^. Galvanostatic charge-discharge (GCD) tests, as well as cyclic stability studies, were performed on an Arbin BT-2000 system in a potential window of 0.05–1.6 V at current densities in the range of 60–1200 mA g^−1^. The specific capacitance is calculated using the formula: C = (I × Δt)/(m × ΔV) where I (A) is the applied value of the discharge current, Δt (s) is the discharge time, m (g) is the mass of the electrochemically active material, and ΔV (V) is the used potential window. The energy density (E, Wh kg^−1^) and power density (P, W kg^−1^) were calculated using the following equations: (1) E = [C × (ΔV)^2^]/7.2 and (2) P = (3600 × E)/Δt, where C (F g^−1^) is the specific capacitance, ΔV (V) is the working voltage window, and Δt (s) is the discharge time obtained from the GCD measurements. The calculated energy and power density values were further used to construct a Ragone plot, enabling evaluation of the energy-storage capability and power delivery performance of the assembled asymmetric supercapacitor [24].

## Figures and Tables

**Table 1:** Elemental composition (wt.%) of K10 and modified samples obtained by XRF analysis.

Sample	Chemical Composition [wt.%]											
	Al	Si	Ca	P	S	Cl	K	Ti	Fe	Mn	Ag	Impurities
K10	11.14	67.73	1.3	0.54	0.13	0.16	6.98	1.50	10.41	—	—	0.11
K10_YP80F	9.46	63.33	1.73	0.76	0.38	0.31	8.14	2.16	12.96	0.06	—	0.71
K10_MnO_2_	7.35	37.42	0.63	0.31	0.14	0.17	4.75	0.71	5.97	42.40	—	0.15
K10_YP80F_MnO_2_	6.47	38.21	0.86	0.45	0.41	0.18	5.50	0.85	5.10	41.00	—	0.12
K10_YP80FAgNPs	11.99	68.01	1.24	0.9	0.41	0.48	5.72	1.44	8.97	0.1	0.54	0.20

**Table 2:** Elemental composition of the synthesized samples determined by EDX analysis (wt.%).

Sample	Chemical Composition [wt.%]										
	C	O	Mg	Al	Si	K	Ca	Ti	Fe	Ag	Mn
K10	7.74	50.39	0.76	6.42	30.88	1.04	0.20	0.25	2.32	—	—
K10_YP80F	76.96	14.12	0.13	1.30	6.81	0.25	—	—	0.43	—	—
K10_MnO_2_	6.77	42.39	0.60	4.88	22.74	1.43	0.17	0.21	1.66	—	19.15
K10_YP80F_MnO_2_	76.32	18.08	—	0.65	2.40	0.21	—	—	0.16	—	2.18
K10_YP80FAgNPs	76.54	14.80	0.14	1.55	6.07	0.36	—	—	0.42	0.11	—

**Table 3:** Equivalent circuit parameters obtained by the fitting procedure.

Sample	Rsl Ω	Rmel Ω	CPE F sn^−1^	n	Rd Ω	Wo-T s	Wo-P
K10_YP80F	0.95	4.97	965	0.78	2.89	0.28	0.49
K10_YP80F_MnO_2_	1.22	0.28	275	1	1.87	0.16	0.49
K10_MnO_2_	1.23	1.61	565	0.8	2.91	0.23	0.49
K10_YP80FAgNPs	1.10	3.41	293	0.871	0.42	0.05	0.46

## Data Availability

The data that support the findings of this study are available within the articles.
